# Evaluating EEG neurofeedback in sport psychology: a systematic review of RCT studies for insights into mechanisms and performance improvement

**DOI:** 10.3389/fpsyg.2024.1331997

**Published:** 2024-07-19

**Authors:** Ming-Yang Cheng, Chien-Lin Yu, Xin An, Letong Wang, Chi-Lun Tsai, Fengxue Qi, Kuo-Pin Wang

**Affiliations:** ^1^School of Psychology, Beijing Sport University, Beijing, China; ^2^Department of Physical Education and Sport Sciences, National Taiwan Normal University, Taipei City, Taiwan; ^3^Department of Sport Psychology, Faculty of Sport Science, Universität Leipzig, Leipzig, Germany; ^4^Sports, Exercise and Brain Sciences Laboratory, Beijing Sport University, Beijing, China; ^5^Center for Cognitive Interaction Technology, Bielefeld University, Bielefeld, Germany; ^6^Neurocognition and Action - Biomechanics Research Group, Faculty of Psychology and Sports Science, Bielefeld University, Bielefeld, Germany

**Keywords:** EEG biofeedback, mental training, sport neuroscience, sport performance enhancement, brain waves, brain training, sport performance, psychomotor efficiency

## Abstract

Electroencephalographic Neurofeedback Training (EEG NFT) aims to improve sport performance by teaching athletes to control their mental states, leading to better cognitive, emotional, and physical outcomes. The psychomotor efficiency hypothesis suggests that optimizing brain function could enhance athletic ability, indicating the potential of EEG NFT. However, evidence for EEG-NFT’s ability to alter critical brain activity patterns, such as sensorimotor rhythm and frontal midline theta—key for concentration and relaxation—is not fully established. Current research lacks standardized methods and comprehensive studies. This shortfall is due to inconsistent EEG target selection and insufficient focus on coherence in training. This review aims to provide empirical support for EEG target selection, conduct detailed control analyses, and examine the specificity of electrodes and frequencies to relation to the psychomotor efficiency hypothesis. Following the PRISMA method, 2,869 empirical studies were identified from PubMed, Science Direct, Web of Science, Embase, CNKI, and PsycINFO. Thirteen studies met the inclusion criteria: (i) proficient skill levels; (ii) use of EEG; (iii) neurofeedback training (NFT); (iv) motor performance metrics (reaction time, precision, dexterity, balance); (v) control group for NFT comparison; (vi) peer-reviewed English-language publication; and (vii) randomized controlled trial (RCT) design. Studies indicate that NFT can enhance sports performance, including improvements in shooting accuracy, golf putting, and overall motor skills, as supported by the psychomotor efficiency hypothesis. EEG NFT demonstrates potential in enhancing sports performance by optimizing performers’ mental states and psychomotor efficiency. However, the current body of research is hampered by inconsistent methodologies and a lack of standardized EEG target selection. To strengthen the empirical evidence supporting EEG NFT, future studies need to focus on standardizing target selection, employing rigorous control analyses, and investigating underexplored EEG markers. These steps are vital to bolster the evidence for EEG NFT and enhance its effectiveness in boosting sport performance.

## 1 Introduction

Mental factors play a crucial role in athletic success, contributing to between 40 and 90% of performance outcomes in sports (Krane and Williams, 2006). Research indicates that skilled athletes can maintain a “cool and focused” brain state by suppressing irrelevant neural activity during the preparatory phase (Del Percio et al., 2009; Bertollo et al., 2016). This ability, known as neural efficiency, reflects a specialized neural process where optimal focus is achieved by minimizing unnecessary neural activities (Milton et al., 2007; Doppelmayr et al., 2008). Furthermore, this concept extends into the realm of sports as psychomotor efficiency, which emphasizes the most effective movement through refined neural processes (Hatfield et al., 2020). Psychomotor efficiency involves the selective activation of task-relevant neural processes and the inhibition of those that are task-irrelevant, indicating that high-level performers fine-tune their neural processes toward achieving a state of automaticity (di Fronso et al., 2016). This streamlined approach to neural and motor coordination underscores the importance of mental training in achieving sports excellence (Callan and Naito, 2014).

Electroencephalography (EEG) is a prominent non-invasive technique for investigating the psychomotor efficiency hypothesis (Hatfield et al., 2020), essential for studying neural mechanisms vital for sport performance during training (Cheng and Hung, 2020a,b) and competition (di Fronso et al., 2019). This research has concentrated on four EEG markers to comprehend psychomotor efficiency in athletes: frontal midline theta (FM theta), left temporal alpha (T3 alpha), sensorimotor rhythm (SMR), and the coherence between left temporal and frontal areas (Chang and Hung, 2020). FM theta has been associated with enhanced and flexible attention among experts (Chuang et al., 2013; Kao et al., 2013). T3 alpha is linked to verbal analytical thinking but shows variable impacts on performance (Parr et al., 2021). SMR is related to the capability to ignore distractions specific to the task and sustain concentration (Cheng et al., 2015b; Wang et al., 2019). Coherence between frontal and left temporal regions is indicative of conscious control (Deeny et al., 2003, 2009; Gallicchio et al., 2017; Lo et al., 2019), suggesting that neural interconnectivity is key in reducing cognitive interference, critical for lessening verbal-analytic distractions (Deeny et al., 2009; Chang and Hung, 2020; Parr et al., 2021). These indicators underscore various neural functions that support psychomotor efficiency, with T3 alpha and FM theta focusing on the activation of pertinent processes, and SMR and coherence on the suppression of irrelevant ones (Deeny et al., 2009; Gallicchio et al., 2016; Cheng et al., 2017). However, the relationship between EEG and the psychomotor efficiency hypothesis remains underexplored in current literature. This gap signifies a need for systematic investigation to elucidate the neural mechanisms underlying sport performance, as proposed by the psychomotor efficiency hypothesis. Neurofeedback training emerges as a promising candidate to address and verify these gaps.

EEG neurofeedback training (NFT) combines neuroscience and sports science to help athletes control their mental states and improve performance using EEG. This method employs operant conditioning to alter brainwave patterns, aiming to boost cognitive, emotional, and motor abilities essential for athletic excellence. Gruzelier et al. (2014) examined the effects of neurofeedback training (NFT) and heart rate variability (HRV) training on 64 contemporary dance students, finding no significant improvements in overall dance performance. Yet, the study highlighted specific benefits: HRV training notably reduced anxiety and enhanced dance technique and artistry, while alpha/theta NFT training increased cognitive creativity. This underscores the importance of psychophysiological interventions in dance education for enhancing particqular aspects of performance and cognitive processing. Moreover, a meta-analysis by Xiang et al. (2018) confirmed NFT’s effectiveness in modifying EEG patterns and positively influencing motor behavior, crucial for athletes. However, a recent meta-analysis by Onagawa et al. (2023) presented inconclusive evidence on NFT’s impact on motor performance metrics such as speed, accuracy, and hand dexterity, primarily due to the small sample sizes of the studies reviewed. Despite its potential in cognitive enhancement and psychiatric contexts, the precise benefits of NFT for sports performance and its mechanisms warrant further exploration. The field confronts challenges like inconsistent methodologies and a lack of theoretical foundation, emphasizing the necessity for standardized research protocols. Additionally, the experiential learning aspects of NFT and their effects are often overlooked (Cheng and Hung, 2020a,b). Establishing standardized protocols would facilitate a more direct comparison of research findings and their practical application. The academic community has called for more empirical research to fill these knowledge gaps (Vernon, 2005; Cooke et al., 2018).

Although current research highlights the potential of NFTs to enhance sport performance, there remains a need for further investigation. Specifically, systematic research is required to understand its impact on psychomotor efficiency. The hypothesis posits that optimal sports performance is contingent upon the precise neural regulation of movements (Hatfield and Hillman, 2001; Hatfield et al., 2020). It is suggested by the psychomotor efficiency hypothesis that individuals with higher skill levels manage their cognitive resources more effectively. As proficiency escalates, the neurological effort required for movement control diminishes, permitting the reallocation of cognitive resources to improve other facets of performance. Empirical studies have established a significant relationship between neurocognitive efficiency and decreased alpha power coherence across temporal and frontal regions (Deeny et al., 2009). Additionally, advancements in neurocognitive state are marked by reduced activity in the sensorimotor cortex, indicated by heightened SMR power (Chueh et al., 2023). This cognitive economy facilitates superior sports performance, highlighting the crucial role of cognitive efficiency in skill development (Cheng et al., 2023). Presently, NFT research in the sports domain primarily focuses on training markers without adequately linking these to performance enhancement (Gong et al., 2021). Such oversight omits vital theoretical contributions like the psychomotor efficiency hypothesis (Hatfield et al., 2020), overlooking three essential elements for enhancing sports performance: identifying brain mechanisms that underpin expert performance, augmenting neural efficiency for sports-related tasks, and examining how modifications in brain activity influence performance outcomes. Therefore, a systematic review of NFT research with an emphasis on psychomotor efficiency is critical to ascertain its effects on sports performance. Adopting this method is pivotal in developing evidence-based training protocols tailored to the unique requirements of athletes across different sports, promising significant practical advantages for both coaches and athletes (Onagawa et al., 2023).

This review seeks to pinpoint EEG markers that signify improved psychomotor efficiency by analyzing randomized controlled trials (RCTs). It focuses on understanding how NFT affects psychomotor efficiency in sports, identifying key EEG markers, and establishing a basis for practical use. Considering previous meta-analyses on sports performance, reevaluating the current research is crucial for several reasons. First, this systematic review aims to elucidate the hypothesis of psychomotor efficiency and its correlation with NFT in sports. This perspective may diverge from the purely quantitative synthesis of existing data. Second, prior analyses have highlighted the issue of insufficient data inclusion (Mirifar et al., 2017; Xiang et al., 2018), a critical factor for ensuring statistical validity. This concern is echoed by the recent meta-analysis conducted by Onagawa et al. (2023), which noted a lack of adequate sample sizes in RCTs investigating NFT’s impact on sports performance. Finally, the issue of outcome measure variability presents a significant challenge. Studies on NFT in sport often report their findings in diverse formats, with some detailing behavioral outcomes and concurrent EEG changes, while others do not. This heterogeneity complicates the task of synthesizing results across studies.

The review sets three aims: first, to meticulously categorize the current literature on EEG NFT in sport, pinpointing existing research gaps. Second, it addresses the challenges hindering NFT research progress, a vital intersection of cognitive neuroscience and sports psychology aimed at enhancing sport performance. Lastly, it proposes a robust pathway that integrates applied and theoretical paradigms, thereby substantially refining training methodologies.

## 2 Research method

### 2.1 Database search

This study conforms to the Preferred Reporting Items for Systematic Reviews and Meta-Analyses (PRISMA) statement. Our search methodology encompassed a thorough review of multiple databases: PubMed, Science Direct, Web of Science, Embase, CNKI, and PsycINFO. We targeted peer-reviewed articles published in English from January 1, 1990, to October 30, 2023. This strategy was deliberately chosen to capture relevant literature within our research scope and to maintain consistency with meta-analytical protocols used in related fields.

Two researchers, CM and AX independently conducted the searches. Our search strategy revolved around three core concepts: EEG, NFT, and sports performance. We linked related terms within each category using “OR” and combined different categories with “AND” to refine the search with no filters. Both acronyms and full terms (e.g., “EEG” and “Electroencephalography”) were included. We supplemented our search with a manual review of references from the identified articles to ensure comprehensive coverage.

### 2.2 Study selection

The article selection process was bifurcated. In the initial phase, CM and AX separately screened titles and abstracts to eliminate irrelevant studies. The second phase involved full-text reviews against a set of predetermined inclusion criteria.

This review employs the PICO framework (population, intervention, control, and outcomes) to outline our study selection strategy (Sackett, 1997), focusing on adults aged 18–64 with proficient skill levels. The intervention scrutinized within this study is NFT, which utilizes various protocols aimed at augmenting or modifying brain activity to enhance motor performance. These motor performances include reaction time, precision, dexterity, and balance. A control group, receiving either no treatment or sham control, is a comparator to isolate and measure NFT’s impact on motor performance. Our primary outcome lies in quantifiable enhancements in motor performance, offering an evaluation of NFT’s effectiveness. This selection strategy is predicated on addressing the core question: “How does NFT, compared to no treatment or sham control, affect motor performance in adults aged 18–64 with proficient skill levels, specifically in terms of reaction time, precision, dexterity, and balance?”

Therefore, the criteria for inclusion in our review are defined as follows:(i) participants aged 18–64 with proficient skill levels; (ii) incorporation of EEG; (iii) administration of NFT training (NFT); (iv) motor performance assessed in terms of reaction time, precision, dexterity, and overall balance; (v) inclusion of a control group for NFT comparison; (vi) publication in a peer-reviewed English language journal; (vii) employment of a randomized controlled trial (RCT) design.

### 2.3 Quality assessment

CM and AX independently assessed the risk of bias in the selected studies using the “Risk of Bias 2.0 (RoB2.0)” tool developed by the Cochrane Collaboration (Higgins and Altman, 2008). This evaluation tool covers five key areas: (1) bias from the randomization process; (2) bias due to deviations from intended interventions; (3) bias from missing outcome data; (4) bias in measuring outcomes; and (5) bias in reporting results. Each domain was classified as having a low, high, or unclear risk of bias, following the guidelines in Chapter 8 of the Cochrane Handbook (Higgins and Altman, 2008).

### 2.4 Search results

**FIGURE 1:**
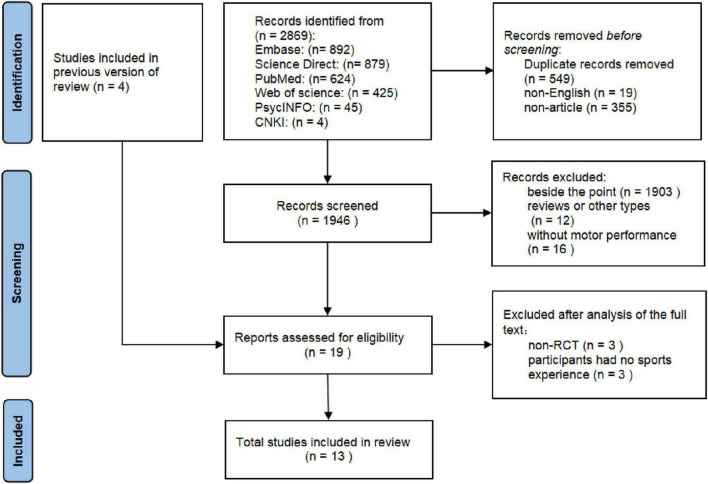
PRISMA flow diagram for study selection.

Using the specified search strategy, we identified 2,869 articles from the database. The search process was completed on October 10, 2023. Of these, 549 were removed due to duplication, and 374 were excluded because they did not meet the language and article type requirements. After reviewing titles and abstracts, 1,931 articles were further excluded for not meeting the inclusion criteria. This process left 19 empirical studies relevant to our topic, which included 4 articles identified from previous reviews. However, 3 of these studies were not conducted using a randomized controlled trial design, and another 3 involved participants without prior exercise training experience. Ultimately, 13 studies satisfied all the inclusion criteria for this systematic review (see Figure 1).

### 2.5 Risk of bias assessment

**FIGURE 2:**
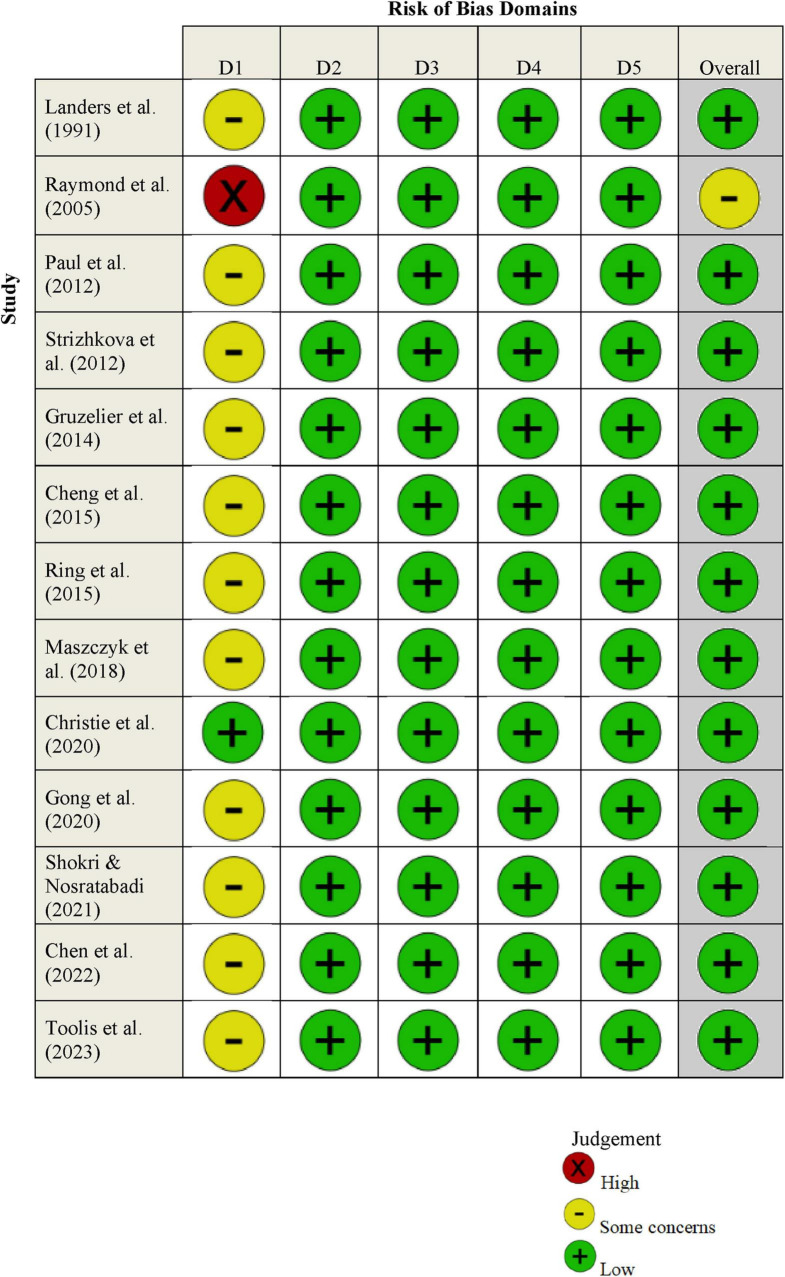
Risk of bias domain. Domains: D1: the process of randomization. D2: deviations from planned interventions. D3: missing outcome data. D4: accuracy in outcome measurement. D5: selection of reported results.

None of the studies evaluated demonstrated a uniformly low risk of bias across all considered domains, except for the research conducted by Christie et al. (2020), as illustrated in Figure 2. A prevalent issue identified was the lack of comprehensive randomization procedures, despite all studies incorporating random grouping of participants. In terms of blinding, only three studies—Landers et al. (1991), Raymond et al. (2005), and Maszczyk et al. (2018)—provided detailed accounts of their blinding processes. Notably, Raymond et al. (2005) and Maszczyk et al. (2018) employed a double-blind methodology. Nevertheless, all studies included in this analysis were assessed to possess a low risk of bias concerning several critical aspects: deviations from intended interventions, incompleteness of outcome data, the accuracy of outcome measurement, and the selection of reported results.

## 3 A comprehensive classification of current NFT literature and identification of gaps

In the exploration of the psychomotor efficiency hypothesis, our investigation employs four key EEG markers to measure psychomotor efficiency in athletes, as identified by Cheng et al. (2023). These markers are FM theta, T3 alpha, SMR, and the coherence between the left temporal and frontal brain areas. Each marker serves to reflect distinct neural mechanisms that are critical for psychomotor efficiency. These studies are systematically categorized based on the specific EEG markers they target for NFT.

### 3.1 FM theta training

FM theta activity is closely linked to sustained attention during the preparatory phase of tasks, playing a crucial role in enhancing performance (Doppelmayr et al., 2008). his relationship is pivotal to the psychomotor efficiency hypothesis, which posits that adaptive neural processing correlates with enhanced behavioral outcomes and superior performance levels (Bertollo et al., 2016). This concise explanation highlights the critical role of FM theta activity in bolstering attention mechanisms pivotal for advanced motor skills.

Toolis et al. (2023) examined the influence of FM theta NFT on rifle shooting performance. Their research found that experienced biathletes could regulate their FM theta activity through NFT, using auditory feedback to potentially improve shooting accuracy and speed by enhancing attentional focus. However, the study also noted that increased FM theta activity did not consistently lead to better real-world shooting outcomes, suggesting a complex relationship between NFT and performance enhancement.

Chen et al. (2022) introduced a novel NFT protocol, the function-specific instruction (FSI) method, aimed at optimizing FM theta, to enhance sport performance. This approach, which integrates personalized verbal instructions with EEG feedback, demonstrated improved FM theta regulation and golf putting performance among skilled golfers. Despite promising results, the study’s focus on skilled golfers limits its general applicability, highlighting the need for additional research to explore the lasting effects of the intervention, its applicability across different sports and skill levels, and the relationship between FM theta activity changes and performance improvements.

The recent studies by Chen et al. (2022) and Toolis et al. (2023) contribute significantly to our understanding of how NFT can influence FM theta to potentially enhance sport performance. Toolis et al. (2023) demonstrated that biathletes with experience could utilize NFT to adjust FM theta activity, which might improve both their shooting accuracy and speed. Nonetheless, the impact of NFT on actual shooting performance in real-life situations is still unclear. On the other hand, Chen et al. (2022) introduced an FSI NFT protocol, revealing its capability to improve FM theta regulation and golf putting performance in skilled golfers. FM theta activity is known to be linked with increased and more flexible attention in experts, suggesting its relevance in sports performance. The integration of training with actual performance, as endorsed by Gong et al. (2021), is highlighted as beneficial, mirroring Chang and Hung (2020)’s call for personalized neurofeedback training (NFT) research. They suggest incorporating competitive stress into NFT sessions and conducting tailored EEG assessments. This strategy focuses on customizing NFT protocols, which could significantly improve training efficiency within limited timeframes, potentially transforming sports training paradigms.

### 3.2 T3 alpha training

Psychomotor efficiency hypothesis posits that diminished activity in specific brain regions during preparatory phases reduces distractions, enhancing performance (Hatfield et al., 2020). A notable aspect of this hypothesis is the association of increased T3 alpha power with reduced reliance on verbal-analytical thinking during physical activities, implying a more efficient neural basis for psychomotor performance.

Strizhkova et al. (2012) discovered that NFT could improve gymnasts’ abilities by increasing T3 alpha power, corroborating the psychomotor efficiency hypothesis by illustrating that specific neural modifications can boost physical performance. Moreover, Strizhkova’s research elucidates NFT’s potential in improving sports performance, including enhanced movement memorization and balance, through brain activity pattern alterations. Earlier research by Kerick et al. (2004) found higher T3 alpha power before starting a movement is a sign of expertise in shooting sports, suggesting skilled marksmen can fine-tune their brain function to focus more on the physical task at hand. This supports the hypothesis by showing how specific interventions can lead to significant improvements in how athletes perform.

Recent studies challenge the straightforward link between T3 alpha power in the brain and sport performance, suggesting a more complex relationship. Parr et al. (2021) and Cheng et al. (2023) highlight that the association between T3 alpha power and performance might vary among different sports and individuals, emphasizing the complex nature of attention allocation during motor tasks (Cooke et al., 2015). This complexity has shifted research focus toward alpha activity in the brain’s central region, which may offer insights into how attention is managed by skilled athletes. For example, Cooke et al. (2015) discovered that highly skilled golfers show lower alpha power at Cz, indicating more effective attention management during motor planning, a finding echoed by Wang et al. (2019) in a study on golf putting. This evolving understanding calls for further exploration into how T3 alpha activity relates to athletes’ use of self-talk, a technique to focus attention during performance. In exploring the influence of self-talk on sport performance, recent research by Bellomo et al. (2020) and Parr et al. (2021) delves into how task-related and unrelated self-talk can affect T3 alpha power, demonstrating that these effects are varied. This suggests that NFT, while promising for improving sport performance, may yield results that are significantly tailored to the individual and the specific sport.

### 3.3 SMR training

Combining research from Cheng et al. (2015a), Gong et al. (2020), and Shokri and Nosratabadi (2021) with the psychomotor efficiency hypothesis reveals how NFT can improve sport performance by optimizing neurocognitive functions. The hypothesis suggests that top motor skills result from better neural efficiency, mainly by minimizing unnecessary sensorimotor cortex activity during detailed tasks (Hatfield et al., 2020). This decrease in excess activity enables sharper and more effective motor performance, consistent with NFT’s positive impacts in sports.

Cheng et al. (2015a) examined the impact of increased SMR power on golf putting performance by recruiting sixteen pre-elite golfers, who were then randomly assigned to either an SMR group or a sham control group. The study found that incorporating NFT into the golfers’ preparation routines significantly improved putting accuracy. This finding supports the psychomotor efficiency hypothesis, illustrating that NFT reduces sensorimotor noise, which in turn enhances focus and efficiency in motor execution. Additionally, the study noted a higher success rate in achieving SMR training targets in the SMR group compared to the control group, marking the first evidence of NFT’s learnability during training. The ease of training reported by the SMR group highlights the adaptive neural processing responsible for the behavioral improvements observed, a concept further elaborated by Bertollo et al. (2016).

Gong et al. (2020) divided 45 healthy participants into three groups to evaluate the impact of NFT on tasks requiring precision, such as shooting. Groups were defined as follows: one received SMR feedback, another received alpha rhythm feedback, and a control group received no neurofeedback training. Over the course of 3 weeks and six training sessions, participants underwent pre- and post-test evaluations measuring shooting performance and changes in EEG resting power. Results highlighted a significant improvement in shooting accuracy for the SMR group, contrasting with a performance decline in the alpha group and no change in the control group. Notably, both neurofeedback groups exhibited marked changes in EEG power post-training, indicative of neuroplastic adaptations. Moreover, the study reported lower perceived training difficulty in the SMR group compared to the alpha group, suggesting the former’s training approach may facilitate easier engagement and potentially greater neurofeedback efficacy. These findings underscore the potential of SMR NFT to enhance performance in precision tasks through neurofeedback, leveraging the brain’s adaptability. Despite these insights, the study does not address the connection between its findings and the psychomotor efficiency hypothesis, leaving a gap in understanding the theoretical underpinnings of how NFT may enhance psychomotor efficiency.

Furthermore, Shokri and Nosratabadi (2021) provided evidence of NFT’s efficacy in basketball, a sport requiring a complex integration of motor and cognitive skills. The comprehensive evaluation underscores the importance of self-regulatory skills facilitated by NFT, which can be interpreted as enhancing psychomotor efficiency through improved neurocognitive processing. This adaptive neural processing, characterized by quiescent activity in the sensorimotor cortex during critical moments of performance, reflects the hypothesis that superior performance is achieved by minimizing irrelevant interference, thereby optimizing attentional focus and motor execution.

In summary, recent studies highlight the positive impact of SMR NFT on sports performance. This training enhances athletes’ concentration and motor skills, leading to greater precision in sports like golf, shooting, and basketball. The research by Cheng et al. (2015a), Gong et al. (2020), and Shokri and Nosratabadi (2021) supports this by showing that SMR NFT helps sport perform more accurately by reducing unnecessary brain activity (Hatfield et al., 2020), which in turn improves focus and execution of movements. For instance, Gong et al. (2020) found significant improvements in shooting accuracy among marksmen who underwent SMR NFT, underscoring the benefits of minimizing distractions for precision-required tasks. Similarly, Cheng et al. (2015a) and Shokri and Nosratabadi (2021) linked NFT to better performance in golf putting and basketball, respectively, supporting SMR NFT’s effectiveness in sports. Additionally, aligning SMR NFT studies with the psychomotor efficiency hypothesis further validates NFT’s contribution to sports enhancement.

### 3.4 Coherence between frontal and temporal region training

The psychomotor efficiency hypothesis emphasizes the importance of Fz-T3 coherence during task preparation, suggesting that optimal motor performance is achieved by minimizing unnecessary cortical activity (Deeny et al., 2009). This reduction in activity helps maintain focus on critical processes required for precision tasks (Hatfield et al., 2020).

Despite the strong link between coherence and psychomotor efficiency in precision sports (Hatfield, 2018), the current research directly targeting coherence as a training target is sparse. Findings indicate that elite marksmen demonstrate heightened focused attention compared to their less skilled counterparts, as shown by lower Fz-T3 coherence in successful shots (Cheng et al., 2023). This suggests that elite marksmen can reduce irrelevant mental distractions, thus maintaining concentration needed for superior performance. Fz-T3 coherence is indicative of the interaction between motor planning and verbal-analytical processing regions, with excessive interaction potentially impairing performance (Deeny et al., 2003; Kao et al., 2013; Wang et al., 2019). Consistent with this, Gallicchio et al. (2017) found that decreased cortico-cortical communication, achieved through practice, enhanced golf putting skills in seasoned golfers, supporting the psychomotor efficiency hypothesis (Hatfield et al., 2020).

The exploration of coherence training in sports presents a significant opportunity for advancement. Kober et al. (2020) investigated the effectiveness of coherence as a target for neurofeedback (NF) training in cognitive tasks. This study engaged twenty adults in ten sessions focusing on modifying EEG coherence between Cz and CPz within the SMR. It examined the influence of SMR coherence adjustment on EEG activity and memory performance. Participants were segregated into groups aiming to either increase or decrease SMR coherence. Enhancements in SMR coherence led to notable shifts in EEG coherence both within and across sessions and a rise in SMR power across sessions, though not within individual sessions. This augmentation correlated with improved memory function post-training. In contrast, efforts to diminish SMR coherence yielded no significant changes in baseline EEG or memory assessments pre- and post-test. These results underscore the viability and potential applications of connectivity-based NF training. Moreover, the protocol to elevate coherence also impacted resting EEG patterns, indicating NF training’s broad effects beyond specific task execution. Remarkably, those undergoing coherence elevation displayed marked advancements in verbal short-term and working memory, implying that augmenting SMR coherence can enhance certain cognitive abilities, potentially by mitigating sensorimotor disruptions (Callan and Naito, 2014).

### 3.5 Training focused on a single target

In a pivotal study by Landers et al. (1991), the impact of NFT on archery performance was rigorously investigated. This study enrolled 24 pre-elite archers in a randomized trial, which was divided into three groups: (a) one receiving accurate feedback to augment low-frequency activity in the left hemisphere, (b) another receiving incorrect feedback aimed at the right hemisphere, and (c) a control group that received no feedback. Participants executed 27 shots in both the pretest and posttest phases, during which EEG were collected from both temporal lobes. The feedback groups underwent NFT, whereas the control group was allotted a 30-min rest period.

The study found that NFT aimed at enhancing slow cortical potentials (SCPs) in the left temporal lobe significantly improved archery performance. Conversely, NFT targeting the right temporal lobe resulted in decreased performance. These findings underscore the potential of NFT to influence performance in precision tasks, such as archery, either positively or negatively, observable after a single session. The research concluded that targeted biofeedback (BFT), informed by established EEG-performance correlations, can selectively boost or hinder the performance of pre-elite archers. Nonetheless, the results did not align with the psychomotor efficiency hypothesis, and subsequent research has not further explored the connection between SCPs and this hypothesis.

### 3.6 Training focused on a single target with other biomarkers

Raymond et al. (2005) explored interventions to enhance dance performance, focusing on the application of alpha-theta NFT and heart rate variability biofeedback. Preliminary findings suggest both alpha/theta NFT and heart rate variability biofeedback potentially enhance dance performance, as judged by evaluators unaware of the treatment conditions. This enhancement was noted in overall execution and specific subscales. Notably, NFT improved “timing,” while heart rate variability biofeedback enhanced “technique.” The significant, albeit modest, improvements underscore the professional relevance of even slight advancements in performance. The findings are contextualized within arousal-performance theories (Hardy and Parfitt, 1991), proposing that biofeedback-mediated physiological regulation may diminish cognitive anxiety and bolster performance, linked to the calming effect of alpha waves and the positive emotional states associated with theta waves. However, the study’s influence is limited by its small sample size and the potential for therapist contact effects due to the lack of an active intervention in the control group.

Gruzelier et al. (2014) also examine the effects of psychophysiological interventions, specifically alpha/theta NFT and heart rate variability training, on contemporary dancers’ performances. The research posits that enhancing the theta/alpha ratio and autonomic regulation through heart rate variability can reduce anxiety and improve dance performance. However, the study acknowledges limitations, including modest intervention effects and potential biases, suggesting a need for future research with a broader participant base and objective measures to validate findings.

Research by Raymond et al. (2005) and Gruzelier et al. (2014) investigates interventions to enhance dance performance, including alpha-theta NFT and heart rate variability biofeedback. Both studies suggest potential improvements in dance performance, with alpha/theta NFT enhancing timing and heart rate variability biofeedback improving technique. Despite the innovative methods and rigorous approach of these studies, they also point out critical shortcomings, such as the necessity for larger participant groups, the inclusion of control groups, and a more profound comprehension of how biofeedback influences performance, consider combining neurocognitive and psychophysiological interventions with additional psychological tactics to create a more comprehensive method for enhancing performance. Addressing the identified deficiencies, such as enrolling larger and more varied cohorts, employing control groups for more valid comparisons, and using objective physiological indicators alongside self-reported metrics, would significantly contribute to this field.

### 3.7 Training focused on multiple targets

Four studies have examined the use of various training targets, with one specifically applying the psychomotor efficiency hypothesis in choosing its targets. This application highlights the critical role of target selection in evaluating effects on behavior, as supported by findings from Mirifar et al. (2017), Xiang et al. (2018), and Onagawa et al. (2023). The varied use of the psychomotor efficiency hypothesis in these studies suggests a potential for improving how targets are chosen for NFT.

#### 3.7.1 Multiple targets (high α, θ)

The study by Ring et al. (2015) delved into the application of NFT within sports, specifically examining its potential to enhance performance by targeting specific brain activity patterns. A critical aspect of their investigation focused on the selection of training targets, particularly the reduction of frontal EEG high-alpha power. This choice was informed by previous research indicating that such brain activity patterns are associated with higher levels of expertise and performance in tasks requiring precise motor control, such as golf putting.

Target selection was fundamentally guided by the psychomotor efficiency hypothesis, which posits that improved performance in motor tasks is related to more efficient neural processing, often evidenced by reduced cortical activity in specific EEG frequency bands like high-alpha power. Babiloni et al. (2008) and Cooke et al. (2015) provided empirical support for this hypothesis, demonstrating that successful putts in golf were associated with decreased high-alpha power over motor and premotor cortical areas. These findings underscored the rationale for targeting high-alpha power reduction in NFT protocols, aiming to emulate the neural efficiency observed in expert performers.

Ring et al. (2015) aimed to improve sports performance by training participants to lower their frontal high-alpha power using NFT, based on the theory that such brain activity reflects the neural efficiency of expert athletes. This method intended to enhance motor planning and execution. The study rigorously evaluated the genuine effects of NFT by establishing a comparison with a sham control group. Participants in the control group underwent an identical protocol to their counterparts in the NFT group, with one critical distinction: the auditory stimulus they received was not reflective of their brain activity. Instead, control group participants listened to pre-recorded tones derived from a participant in the NFT group who was matched with. This approach ensured uniformity in the experiment by turning off the tone an equal number of times and for equivalent durations across both groups. Additionally, it guaranteed that participants in both groups completed a comparable number of putts during the experiment’s acquisition phase. The study measured EEG activity and evaluated putting performance under varying levels of pressure.

Despite expectations, the study’s outcomes were mixed. While the NFT group succeeded in reducing their frontal high-alpha power, demonstrating the ability to influence brain activity, this did not lead to better putting performance compared to the control group. Improvements were similar across both groups, regardless of pressure.

#### 3.7.2 Multiple targets (θ, β)

Maszczyk et al. (2018) investigated the effects of EEG-based NFT on the dynamic balance of judo athletes, an important aspect of sports performance. The research utilized a controlled experimental approach, incorporating sham feedback to validate the results. Participants completed ten NFT sessions, focused on increasing the 14–19 Hz frequency band and decreasing the 3–8 Hz frequency band at the O1 and O2 scalp locations. The findings revealed that the training significantly improved the athletes’ balance.

The selection of specific brainwave frequencies for the training was guided by prior research (Dziembowska et al., 2016), which has shown that biofeedback training can improve heart rate variability, alter brainwave patterns, and enhance the autonomic nervous system’s flexibility, aiding in athletes’ stress management. This study included eighteen judo athletes, divided into a treatment group, which received the actual NFT, and a control group, which received placebo feedback. Post-training, the treatment group displayed notable improvements in dynamic balance, indicating the effectiveness of NFT in improving physical balance. The research documented significant changes in theta, alpha, and beta brainwave frequencies in the treatment group, linking these changes to the improvements in balance.

Nonetheless, the study has certain limitations, such as an unclear rationale for the choice of target frequencies and a lack of comprehensive analysis on the EEG and behavioral changes. The omission of a discussion on the psychomotor efficiency hypothesis indicates potential areas for further investigation.

#### 3.7.3 Multiple targets (SMR, θ, high β)

Christie et al. (2020) study critically evaluated the efficacy of SMR NFT and biofeedback training (BFT) on enhancing ice hockey shooting performance, guided by the psychomotor efficiency hypothesis. The authors hypothesized that improvements in psychomotor tasks can be achieved through optimized brain activity, specifically increased SMR power, which is associated with heightened focus and motor control (Cheng et al., 2017). The objectives were to assess if SMR-NFT/BFT could: (a) improve shooting performance, (b) induce neurological adaptations, and (c) link these adaptations to performance enhancements.

The study found significant performance improvements in the SMR-NFT/BFT group compared to controls, indicating that NFT/BFT could effectively enhance psychomotor efficiency in sports. Participants in the intervention group showed a notable increase in SMR power in lab settings, suggesting neurological adaptations. However, these changes were not observed during actual shooting performance, highlighting a gap in translating lab-based neurological improvements to real-world sports performance.

This study corroborates existing evidence on the effectiveness of NFT and BFT in sports performance enhancement (Beauchamp et al., 2012), while also emphasizing the importance of methodologically rigorous research to clarify the causal mechanisms underlying these benefits (Hung and Cheng, 2018). It underscores the value of controlled, precise studies in advancing our understanding and application of NFT/BFT, highlighting the potential for these interventions to significantly improve motor skills when implemented under well-defined conditions.

However, it also reveals the complexity of translating lab-based neurophysiological adaptations to enhanced performance in competitive sports settings. Further research is required to bridge this gap and fully harness the potential of NFT/BFT in sports performance enhancement.

#### 3.7.4 Multiple targets (SMR, θ, β)

Paul et al. (2012) conducted a study to evaluate the effectiveness of NFT in enhancing the performance of university-level archers. The study specifically examined precision and several psycho-physiological indicators such as heart rate, pleasure, arousal levels, and the sensory motor rhythm (SMR)/theta ratio during competitive situations. This study was predicated on the theory that augmenting the SMR through EEG feedback could enhance concentration and accuracy, which are paramount in archery.

The study reported significant improvements in the archers’ pleasure and arousal levels before competition, arousal levels after competition, and the SMR/theta ratio in the experimental group relative to the control group following a 4-week NFT intervention. These findings indicate that NFT may facilitate better regulation of psychological and physiological states, thereby improving performance metrics.

Despite its rigorous experimental design and quantitative methodology, the study encounters several limitations that necessitate further investigation. The limited and homogenous sample size, along with the lack of in-situ EEG measurements, underscores the need for more comprehensive and varied research efforts. Future studies should aim to explore a wider spectrum of psycho-physiological factors relevant to sports performance. Moreover, an in-depth theoretical exploration is required to elucidate how training the SMR/theta ratio could be optimized to boost athlete performance.

### 3.8 Impact of psychomotor efficiency on NFT utilization in sports

The application of the psychomotor efficiency hypothesis to NFT in enhancing sports performance is identified as a promising but insufficiently examined field. The current body of research presents numerous limitations that compromise its potential effectiveness and reproducibility. The following section systematically addresses these limitations, underscoring the imperative for an improved research methodology to robustly establish NFT’s theoretical and practical foundations in sport.

#### 3.8.1 Inconsistent EEG target selection and misalignment with psychomotor efficiency hypothesis

The selection of EEG targets—high α, θ, β, and SMR—believed to influence psychomotor efficiency, shows notable variability across studies. This inconsistency and the absence of empirical support for their relevance to the psychomotor efficiency hypothesis weaken the foundation of NFT protocols designed to boost sports performance. Cheng and Hung (2020a) underscore this gap, calling for a more uniform approach to target selection and a clear theoretical connection between EEG targets and cortical processing effectiveness in training. Wang et al. (2023) further emphasize the need for rigorous validation of these targets within the framework of the psychomotor efficiency hypothesis. The lack of consistent evidence supporting the hypothesis casts doubts on its correct application in NFT research for sports, challenging its predictive value for performance outcomes (Ros et al., 2020).

#### 3.8.2 Neglected use of coherence as a training target

Research indicates that superior marksmen, compared to their less proficient counterparts, exhibit lower Fz-T3 coherence during successful shots, suggesting a better ability to reduce irrelevant interference and maintain focused attention (Haufler et al., 2000; Deeny et al., 2009). Yet, no study has directly targeted this EEG marker for training, overlooking a potentially significant factor for enhancing shooting performance.

EEG coherence significantly contributes to our understanding of psychomotor efficiency by elucidating the cortical inputs involved in motor planning and execution, notably within the frontal (Fz) and motor cortex (C3, Cz, C4) areas. Zhu et al. (2011) effectively demonstrated that diminished connectivity between the left temporal region (T3) and Fz is associated with superior performance in golf putting task. This observation is consistent with Fitts and Posner (1967) autonomous stage theory, suggesting that reduced neural connectivity leads to decreased interference in motor planning, thereby enhancing performance efficiency.

Adding to this, Gentili et al. (2015) investigated the changes in cortical dynamics during cognitive-motor adaptation tasks, revealing that EEG coherence between the Fz and left hemisphere decreases progressively across trials (spanning low-beta, high-beta, and gamma bands), coinciding with improvements in the speed and accuracy of movements. Notably, a significant reduction in coherence across delta, low/high-theta, and low/high-alpha bands between Fz and the left prefrontal region indicates a gradual withdrawal of frontal executive control. This shift away from active engagement in established visuomotor patterns to a more autonomous processing mode is manifested through enhanced motor performance, characterized by faster, more direct movements, and reduced error rates in later adaptation phases (Callan and Naito, 2014; Bertollo et al., 2016; di Fronso et al., 2016).

These studies underscores the significance of EEG coherence in elucidating the neural underpinnings of the psychomotor efficiency hypothesis. By examining cortical connectivity patterns and selecting coherence as the focal point for NFT in sports, researchers could unlock insights into the neural integration processes essential for executing psychomotor tasks.

#### 3.8.3 Need for comprehensive control analysis

To Control analyses are crucial for determining the efficacy of NFT. These analyses include addressing electrode specificity, frequency specificity, and sessional learning effects before and after NFT application (Cheng et al., 2015a). Utilizing sham control groups is vital for an accurate evaluation of NFT’s actual effects, thus rigorously assessing any potential placebo impacts. Further essential aspects for control include examining NFT’s retention effects, the integrity of instructions provided, and the documentation of behavioral and cognitive outcomes on an individual basis. Variability in outcomes can be attributed to factors such as the specific condition being treated, the participant’s engagement with the NFT process, and the particular protocol used, underscoring the importance of quantifying individual differences to validate NFT’s applicability.

Table 1 summarizes the reviewed studies. In summary, NFT in sports, guided by the psychomotor efficiency hypothesis, has shown potential in improving sport performance. However, the current body of research reveals significant inconsistencies in the selection of EEG targets, alignment with the hypothesis, and validation of outcomes. Studies demonstrate varying effectiveness of NFT in sports performance, with some showing promise in specific tasks like shooting and golf putting. The selection of appropriate EEG markers for NFT is crucial, as different markers may yield varying results depending on the sport and the athlete’s skill level. Despite the potential of coherence training in precision sports, limited research has explored this area. To establish solid empirical evidence, robust control studies are essential to validate NFT interventions, considering factors like providing the theoretical and evidence-based selection of electrode placement, frequency selection, and investigating the within-session learning effects. Future research should aim to clarify these issues and establish guidelines for NFT protocols in sports to improve sport performance.

**TABLE 1:** List of randomized controlled trials investigating sport performance enhancements.

No.	References	Motor task	Frequency	Duration	Training target	Feedback type	Task of control group	Feedback instruction	Functional training
1	Landers et al., 1991	Archery	/	/	Single (low potential)	Visual	Without any intervention	Unknown	Training integrated with performance
2	Raymond et al., 2005	Dance	/	4 weeks	Single (α/θ)	Audio	Without any intervention	Specific	Training w/o performance integration
3	Paul et al., 2012	Archery	3/wk	4 weeks	Multiple (SMR, θ, β)	Both	Without any intervention	General	Training w/o performance integration
4	Strizhkova et al., 2012	Ideomotor execution	1/d	/	Single (α)	Audio	Without any intervention	Specific	Training w/o performance integration
5	Gruzelier et al., 2014	Dance	2/wk	12 weeks	Single (α/θ)	Audio	Without any intervention	Specific	Training w/o performance integration
6	Cheng et al., 2015a	Golf putting	2/wk	5 weeks	Single (SMR)	Both	Mock feedback	Specific	Training integrated with performance
7	Ring et al., 2015	Golf putting	/	/	Multiple (high α, θ)	Audio	Sham	General	Training integrated with performance
8	Maszczyk et al., 2018	Dynamic balance	/	5 weeks	Multiple (θ, β)	Both	Sham	Unknown	Training w/o performance integration
9	Christie et al., 2020	Ice hockey shooting	/	4.5 months	Multiple (SMR, θ, high β)	Visual	Without any intervention	Unknown	Training w/o performance integration
10	Gong et al., 2020	Pistol shooting	2/wk	3 weeks	Single (SMR)	Both	Without any intervention	General	Training w/o performance integration
11	Shokri and Nosratabadi, 2021	Basketball performance	3/wk	8	Multiple (SMR, α, θ)	Audio	Without any intervention	Unknown	Training integrated with performance
12	Chen et al., 2022	Golf putting	/	/	Single (FMT)	Audio	Sham	Specific	Training integrated with performance
13	Toolis et al., 2023	Rifle shooting performance	/	/	Single (FMT)	Audio	Without any intervention	General	Training integrated with performance

## 4 Issues to advancing EEG NFT research

Despite advancements in NFT research, unresolved challenges continue to impede its further development. Addressing these issues is crucial for advancing NFT and its application.

### 4.1 Target selection

The variation in outcomes observed in current research highlights the critical need for a data-driven approach in selecting training targets (Onagawa et al., 2023). It is advised that NFT targets adhere to evidence-based protocols to significantly enhance sports performance, with target selection grounded in a solid theoretical framework and validated by prior research (Cooke et al., 2018). It is posited that the neuronal mechanisms underlying performance enhancement through NFT vary across different outcomes. Identifying specific NFT targets with quantitative electroencephalography assessments is critical for addressing different performance aspects in sports (Enriquez-Geppert et al., 2017).

Without personalized target selection and clear rationale, NFT may not effectively enhance neural networks related to sport performance, leading to suboptimal outcomes. Jeunet et al. (2019) highlight the importance of applying EEG-based brain-computer interfaces and NFT focusing on SMR to improve motor skills. They also point out the current evidence’s lack of a consistent rationale for target selection, which complicates the link between SMR BCI/NFT and motor skill enhancement. This inconsistency underlines the need for precise target selection in NFT interventions to avoid unclear results and establish NFT as a standardized method for enhancing motor skills in sports.

In summary, the variability in outcomes of previous studies can be linked to differences in training targets (Mirifar et al., 2017; Xiang et al., 2018). Future research should adopt a data-driven approach for selecting training targets to improve ecological validity, including a clear rationale for target selection (Cooke et al., 2018; Hung and Cheng, 2018). This rigorous methodology will advance NFT research in sports, ensuring logical, thorough, and relevant findings with real-world applicability.

### 4.2 Basis of psychomotor efficiency hypothesis

Neurofeedback training has been effectively used to enhance sports performance, suggesting a significant link with psychomotor efficiency. Such adaptive neural processing leads to effective behavioral outcomes and superior performance (Bertollo et al., 2016). This hypothesis, as outlined by Hatfield et al. (2020), suggests that exceptional motor performance is fundamentally supported by specialized neurocognitive processes. These processes are instrumental in the strategic allocation of cortical resources, thereby optimizing performance outcomes. A critical component of this optimization is deploying advanced attentional strategies, essential for achieving proficient performance in sports (Bertollo et al., 2016) that demand precise motor execution, such as golf putting. These strategies incorporate adaptive neurocognitive processes, enabling skilled performers to attain superior performance levels (Wang et al., 2021; Chueh et al., 2023).

In the current reviewed research, only Christie et al. (2020) directly discusses their findings in the context of the psychomotor efficiency hypothesis, while several studies have investigated its key markers (Cheng et al., 2015a; Gong et al., 2020; Shokri and Nosratabadi, 2021; Chen et al., 2022; Toolis et al., 2023). Notably, an emerging body of evidence highlights the efficacy of NFT in improving motor performance, with significant advancements observed following just a single training session. This is particularly evident in the work of Chen et al. (2022), Kao et al. (2014), and Wang et al. (2023), which collectively underscore the symbiotic relationship between NFT and psychomotor efficiency through the demonstration of enhanced motor performance post-training.

Chueh et al. (2023) investigate the impact of psychomotor efficiency on skilled golfers by analyzing the relationship between specific EEG indices—FM theta, left temporal alpha, SMR, and frontocentral alpha power (FCα)—and the success rate of golf putting. This examination involved collecting and analyzing EEG from twenty-seven skilled golfers during both successful and unsuccessful putting attempts, employing mixed-effects logistic regression for data analysis. The findings indicate a significant association between increased FM theta and left temporal alpha powers with a higher likelihood of successful putting. This underscores the critical role of refined attention allocation in enhancing golf putting performance. Additionally, elevated SMR and FCα activities were correlated with improved performance, highlighting the importance of efficient motor program processing. These results provide valuable contributions to the existing literature by illustrating how cognitive-motor performance in golf can be enhanced through specific EEG indices activation, as supported by Chen et al. (2019) and Wang et al. (2021). The study’s implications for developing training regimens that focus on improving psychomotor efficiency in skilled golfers are substantial, resonating with the methodologies and findings of related research (Cheng et al., 2023).

In summary, Chueh et al. (2023) provide robust evidence emphasizing the importance of precise activation and control of specific brain regions for optimal cognitive-motor functioning in sports, particularly those demanding high accuracy. This work bolsters the psychomotor efficiency hypothesis proposed by Hatfield (2018), underlining its applicability in enhancing sports performance through NFT. These specific brain areas are essential for athletes to concentrate on pertinent tasks while filtering out distractions, highlighting the critical role of cognitive elements in improving sports performance (di Fronso et al., 2016). These outcomes are further supported by studies leveraging the multi-action plan (MAP) model (Bertollo et al., 2016). Such research advocates for the strategic allocation of critical cortical resources to optimize performance, as discussed by di Fronso et al. (2016), suggesting future investigations to further delineate the neurocognitive mechanisms underlying.

### 4.3 Validation of training effect

Neurofeedback training in sports performance has drawn attention to the necessity of evaluating the specificity of training effects and exploring its long-term impacts on athletes. The precision in targeting specific brain regions is fundamental to the efficacy of NFT. However, its broad application could inadvertently affect non-targeted areas, potentially diluting the intended training outcomes. This highlights the essential role of meticulous control analyses in ensuring that performance enhancements are directly attributable to the intended training interventions (Vernon, 2005; Morrone and Pedlar, 2024). It emphasizes the necessity for specificity in training approaches, including the valid control group(s) and the assessment of long-term effects (Hung and Cheng, 2018).

The generalized approach of NFT in modulating brain activity may inadvertently influence unintended brain regions or functions. Gruzelier (2014) identified three critical factors for NFT’s specificity: feedback impact, feedback frequency band, and feedback location. The occurrence of “entrainment,” where unintended changes in frequency bands and brain regions are observed, questions NFT’s precision. This challenge necessitates rigorous control analyses to ensure that observed improvements are directly linked to the specific training goals. The difficulty in achieving training specificity and its implications for NFT’s outcomes are further evidenced by research from Cheng et al., 2015a,Chen et al. (2022), and Wang et al. (2023), which performed detailed analyses on frequency and topographical specificity.

Positioning NFT as a promising candidate for long-term enhancement in sport performance, the research conducted by Pourbehbahani et al. (2023) sheds light on its lasting benefits. The study explored the effects of SMR NFT and self-controlled practice on motor learning in novice golfers, finding both methods independently improved performance, particularly in acquisition and post-test stages. However, only SMR NFT showed sustained benefits in follow-up, also enhancing SMR power. The investigation calls for a prudent approach, emphasizing that the long-term effects of NFT necessitate thorough research to completely comprehend its impact. This includes the critical step of integrating an active control group and the assessment of key variables such as intrinsic motivation and positive emotions. These factors are essential for unlocking the mechanisms of self-regulation practices that NFT can potentially enhance. Such meticulous evaluation is crucial for identifying how NFT can be seamlessly incorporated into strategies designed to improve sports performance. The effects of single-session NFT on sports performance remain debated, with studies varying in training targets, such as frontal midline theta (Chen et al., 2022), mu rhythm (Wang et al., 2023), and SMR power (Wu et al., 2023). Moreover, the effectiveness of NFT is further evidenced by the induced behavioral and neurophysiological changes attributable to successful operant feedback contingencies. These changes have been shown to correlate directly with learning indices and subsequent improvements in performance (Ros et al., 2020), establishing NFT as a potent standalone intervention for sports performance enhancement. Yet, the impact of NFT varies among individuals, with factors such as sensory modality dominance playing a significant role in its efficacy (Kober et al., 2015).

In conclusion, the focus on training specificity and the investigation into the long-term effects of NFT underscore the importance of methodological rigor, theoretical grounding, and empirical validation in enhancing sports performance. This involves critically evaluating its impact on athletes, with specific reference to the psychomotor efficiency hypothesis. Such a detailed evaluation is essential to ensure that the application of NFT is grounded in a robust understanding of its effects and potential outcomes (Gruzelier, 2014; Park et al., 2015; Ros et al., 2020; Wang et al., 2023). This comprehensive approach may support for efficacy in training, the potential for sustained benefits, and the importance of individualized training approaches (Cooke et al., 2018; Cheng and Hung, 2020a).

## 5 Future trajectories in NFT development

### 5.1 Performance-focused NFT research for sport performance enhancement

#### 5.1.1 Integration with physical practice and technology

Merging NFT with physical exercises or Virtual Reality (VR) can render training more pertinent and aligned with real-world applications. Studies have shown that integrating NFT with physical activities can significantly improve visuomotor skills (Cheng et al., 2015a; Shokri and Nosratabadi, 2021; Toolis et al., 2023). Tailoring NFT to meet individual requirements is essential for its effectiveness. Although the combination of VR with specific NFT practices remains relatively unexplored, preliminary evidence indicates that such integration could substantially enhance cognitive control and sport performance. Baqapuri et al. (2021) found that engagement levels in a VR environment were higher, with participants showing progressive improvement in game performance while applying NFT strategies. This synergy between VR and NFT not only underscores the role of virtual environments in augmenting NFT but also suggests a potential for broadening the scope of NFT applications through the facilitation of implicit self-regulation skills, even in the face of the mental fatigue associated with extended NFT sessions. Additionally, the integration of VR is shown to sustain trainees’ interest and prolong the effectiveness of NFT interventions (Baqapuri et al., 2021).

#### 5.1.2 Individualization of NFT

Neurofeedback training research for athletes are encouraged to be conducted in familiar settings to ensure its relevance and applicability to their sports performance. The main objective is to develop straightforward educational strategies that highlight the role of cognitive functions in improving sports performance. It’s crucial for athletes to learn NFT techniques that seamlessly integrate into their daily training routines. Field studies, employing rigorous methodologies with control groups, are necessary to evaluate the practical impact of NFT on performance and to differentiate its effects from placebo responses.

To enhance the efficacy of NFT, it’s important to customize the training to the athlete’s specific sport, performance goals, and baseline EEG data. Customization is key due to the unique cognitive and motor demands of different sports. For example, golfers seeking to improve putting accuracy require NFT that focuses on brain functions related to precision (Xiang et al., 2018; Cheng and Hung, 2020b; Gong et al., 2021). A study on college students using functional near-infrared spectroscopy (fNIRS) for personalized NFT showed enhanced attention and greater activation of the prefrontal cortex compared to standard NFT techniques (Gu et al., 2022). This personalized approach has proven effective in sports-specific training, as evidenced by Chen et al. (2022). Research by Wang et al. (2023) involving thirty novice individuals divided into three groups—function-specific instruction (FSI), traditional instruction (TI), and sham control (SC)—revealed that FSI significantly improved golf putting performance after just one session of EEG-NFT. This finding supports the efficacy of tailored NFT protocols and suggests a link between changes in Mu power and motor control improvements, underscoring the need for further investigation under the psychomotor efficiency hypothesis (Afrash et al., 2023; Wang et al., 2023).

Arns et al. (2008) demonstrated that NFT, tailored to individual EEG profiles and using real-life scenarios, can enhance specific sports skills such as golf putting. This evidence highlights NFT’s capability to improve sport performance via contextually relevant training, suggesting its applicability beyond sports to broader clinical realms. Yet, the inclusivity of NFT research concerning sex and age representation within sports populations remains critical for the validity of such studies.

Future investigations should systematically consider individual differences that influence learning outcomes in NFT. These include the rate of learning, fatigue accumulation during training sessions, variations in motivation, and personality traits. Gruzelier (2014) emphasized that these factors, along with unexplored sex differences, could serve as important guides for customizing training schedules. Moreover, an athlete’s level of expertise may significantly impact NFT’s effectiveness, underscoring the necessity to tailor training protocols to the individual’s skill level (Ring et al., 2015; Wang et al., 2023).

### 5.2 Integration NFT with psychological training

Psychological Skills Training (PST) is key in boosting sports performance by fostering emotional resilience, cognitive skills, and mental toughness. The integration of psychological skills training into athletes’ pre-competition routines is advantageous, as Blumenstein and Orbach (2018) have shown. They propose a method to combine biofeedback training with psychological skills training via the Wingate five-step approach, consisting of introduction, identification, simulation, transformation, and realization phases. Additionally, they enhanced this model with the Learning-Modification-Application strategy, customizing psychological techniques to meet athletes’ specific needs in high-pressure and distraction-filled settings.

Siekanska et al. (2021) conducted a comprehensive review on the role of technology in enhancing psychological skills training for elite athletes. The review emphasizes the significance of psychological strategies such as goal setting, relaxation, and self-regulation. Despite the promising results of integrating technology into psychological skills training, Siekanska et al. (2021) highlight the need for methodological advancements in future studies to develop standardized practices and verify the effectiveness of these technologies.

In essence, psychological skills training offers considerable opportunities to utilize technology in improving sports performance. This method is likely more accessible and familiar to practitioners and coaches compared to newer technological interventions. Aligning with previous suggestions, the use of BFT and NFT to measure the effectiveness of psychological skills training on enhancing stress resistance promises to provide clear, measurable results (Blumenstein and Orbach, 2018; Cheng and Hung, 2020a,2020b).

### 5.3 Combining NFT with imagery

Combining NFT with motor imagery techniques offers an innovative strategy for improving athletic performance, drawing parallels to traditional motor imagery training. This method involves athletes using feedback from visual or auditory stimuli to mentally rehearse and visualize the intricacies of their movements and sensations, potentially enhancing performance outcomes. Integrating NFT with imagery practices has been associated with notable improvements in performance metrics, including enhanced motor imagery, increased brain activity, improved manual dexterity, and reduced response times (Zhuo and Li, 2014). Additionally, there has been a focus on improving the accuracy of real-time brain state classification during motor imagery tasks, which is pivotal for applications in brain-computer interfaces. Bagarinao et al. (2020) investigated the effects of NFT-induced changes in brain activation on the classification of task-related brain states within a motor imagery context. Their research indicated that NFT led to a transition from broad to more localized brain activation. Continuous updates to the classifiers to reflect these changes resulted in significant improvements in accurately classifying motor imagery-related brain states. This progress enhances the functionality of brain-computer interfaces by improving user control. It boosts the efficacy and reliability of NFT, which is crucial for achieving optimal training efficacy.

## 6 Conclusion

Neurofeedback training holds promise for improving sports performance, grounded in the psychomotor efficiency hypothesis. Yet, the field is hampered by significant research gaps, including inconsistent EEG target selection, a shortfall in confirming the impact of training on EEG coherence, and inadequate control analyses for validating the training effect. Future research should aim to standardize target selection, investigate evidence-based EEG markers, and apply rigorous control analyses (Ros et al., 2020). Additionally, more real-world training studies are encouraged to improve ecological validity, which is vital to establish a robust empirical basis for NFT in sports. Addressing these challenges will advance our scientific understanding of NFT’s mechanisms and facilitate its practical application across various athletic disciplines. Bridging these gaps to fully unleash NFT’s potential in optimizing sports performance and training protocols is crucial.

## Author contributions

M-YC: Writing – review and editing, Writing – original draft, Data curation, Conceptualization. C-LY: Writing – review and editing. XA: Writing – review and editing, Writing – original draft, Validation. LW: Writing – review and editing. C-LT: Writing – review and editing, Writing – original draft. FQ: Writing – review and editing, Writing – original draft. K-PW: Writing – review and editing, Writing – original draft.
